# Intramedullary Nailing for Lower Limb Polyostotic Fibrous Dysplasia in Children: A Long-term Follow-up Study

**DOI:** 10.1097/BPO.0000000000002097

**Published:** 2022-02-24

**Authors:** Ernesto Ippolito, Pasquale Farsetti, Roberto Caterini, Enrico Micciulli, Giulio Gorgolini, Laura Ruzzini

**Affiliations:** * Department of Orthopaedic Surgery, University of Rome “Tor Vergata”, Viale Oxford; † Department of Orthopaedic Surgery, Pediatric Hospital “Bambino Gesù”, Palidoro, Rome, Italy

**Keywords:** polyostotic fibrous dysplasia, McCune-albright syndrome, intramedullary nailing, lower limb, children

## Abstract

**Background:**

In children, intramedullary nailing (IN) has been proposed as the best treatment when the femur and tibia are totally affected by fibrous dysplasia (FD). However, in younger children IN must be repeated to maintain stabilization of the affected skeletal segment during growth. We report the long-term results in a cohort of patients in whom more than two-thirds of cases had IN repeated during growth.

**Methods:**

Twenty-nine femurs and 14 tibias totally affected by FD were treated by IN in 21 patients with polyostotic FD and McCune-Albright syndrome. Thirteen patients with 35 femoral and tibial deformities had a painful limp whereas 8 presented fractures. The patients had their first IN at a mean age of 9.26±2.68 years (range: 4 to 14 y). IN was repeated during growth in the younger patients, and all the patients underwent a mean of 2.13 femoral and 1.50 tibial IN per limb. The last IN was performed at a mean age of 16.42±1.95 years (range: 11 to 19 y). Titanium elastic nails and adult humeral nails were used in younger children, whereas adult femoral cervicodiaphyseal and interlocking tibial nails were used in older children and adolescents. At the latest follow-up, the patients were evaluated with a clinicoradiographic scale. All the data were statistically analyzed.

**Results:**

The mean length of follow-up from the last IN was 6.47±3.10 years (range: 3 to 14 y), and the mean age of the patients at follow-up was 22.85±3.53 years (range: 14 to 29 y) when lower limbs were fully grown in all but 1 patient. Satisfactory long-term results were obtained in about 81% of our patients, while complications occurred in 32.5% of the 43 cases.

**Conclusion:**

Lower limb IN—that was repeated in younger children during growth—provided satisfactory long-term results in most of our patients, with fracture and deformity prevention and pain control, regardless of the high rate of complications that mainly affected the femoral cases. Missing scheduled follow-ups was the main predictor of a poor result.

**Level of Evidence:**

Level IV—case series.

Both femur and tibia may be extensively affected in polyostotic fibrous dysplasia (PFD) and McCune-Albright syndrome (MAS), causing pain, fracture and deformity that often lead to the patient’s morbidity and disability since childhood.^1^–^7^

There is nowadays general agreement on the surgical management of lower limb PFD.^3^,^6^–^12^ In adults, intramedullary nailing (IN) appears to be the best surgical treatment^3^,^7^,^13^–^15^ although it has not yet been clearly established in children, in whom early IN is likely to require subsequent revision surgery because the nailed skeletal segment grows and the nail becomes insufficient to hold both the increased size of the bone and the increased weight of the patient.^12^,^16^–^21^

The need for periodic revision surgery in children has already been confirmed by several authors although only expert opinions, case reports and small series of patients with short-term follow-up have been published on femoral IN in children with PFD/MAS.^8^,^9^,^12^,^16^,^17^,^19^–^23^ Moreover, as far as we know, no longitudinal study has been published in children operated on with lower limb IN and followed up to the end of lower limb growth by the same surgical team. The aim of our study is to report the long-term results in a cohort of patients who had repeated IN in two-thirds of the treated cases during growth.

## METHODS

After Institutional Review Board approval was obtained, 27 consecutive patients with either PFD or MAS operated on by femoral and tibial IN were identified from our hospital registry between January 1998 and December 2017. Four patients were excluded because their femurs and tibias were only partially affected by fibrous dysplasia (FD), while 2 patients refused to participate. The remaining 21 patients with the femur and tibia totally affected by biopsy-proven FD—either themselves or their families depending on their age—gave us their informed consent to be included in the study.

All the patients were diagnosed for PFD/MAS at a mean age of 5.85±2.53 years (range: 2 to 12 y), and then referred to our hospital which is a national referral center for their orthopaedic management. Ten patients were male and 11, female; 16 had MAS and 5, PFD. When admitted to our hospital for surgery, 13 patients had a painful limp whereas 8 presented fractures. Nine of the 21 patients have already been reported with a shorter follow-up in 2 of our previous publications when the 9 patients had their first INs either with adult humeral nails (PHN) 16 or as the second stage of a 2-stage procedure.^24^ Indications for surgery were either fractures or a limp caused by an angular/bowing deformity of the femur/tibia or chronic pain without deformity of these same skeletal segments. Before admission to our hospital, 8 patients who complained of diffuse lower limb pain had had intravenous Zoledronic acid administration (0.1 mg/kg), while 7 MAS patients with low phosphoremia and a high serum level of alkaline phosphatase had received 1 µg of 1,25 dihydroxycholecalciferol (calcitriol) per os a day, and 250 mg of sodium phosphate per os 4 times a day.

**FIGURE 1:**
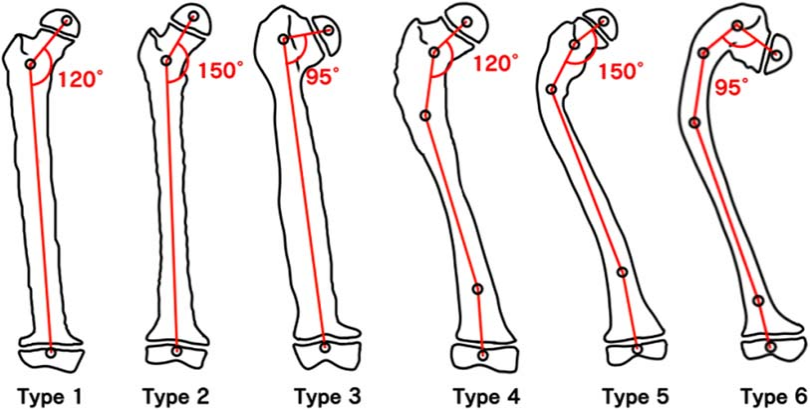
The 6 types of femoral deformities on the coronal plane according to Ippolito et al.^25^

Standing radiographs were done and femoral deformities were classified on the coronal plane according to Ippolito et al^25^ (Fig. 1), whereas the prevalent deformity on the lateral plane was an anterior bowing. There were 6 type 1 deformities; 2 type 3; 9 type 4; 2 type 5; and 6 type 6. Lying radiographs were done in fractured patients. Either medial or anteromedial bowing were the prevalent deformities in the tibia. In type 4, 5, and 6 femoral deformities as well as in tibial deformities, the Malalignment Test of Paley^26^ was performed to identify the apex of the deformity and plan the level of the osteotomy. Multiapical bowing deformities^26^ required more than one corrective osteotomy, while in type 3 and 6 femoral deformities, when the whole proximal femur was entirely involved by FD tissue and the neck-shaft angle was ≤90 degrees, we performed a 2-stage correction.^24^ At the time of IN, FD tissue was never curetted or replaced by either bone grafts or bone cement. In all cases, we performed open osteotomies with a saw and, before IN, intramedullary reaming was performed because long bones totally involved by FD tissue lack a medullary canal.^26^,^27^ After surgery, patients stayed in bed for 3 to 5 days and, as soon as they felt comfortable, partial weight-bearing was allowed with the use of a walker or crutches. When TENs were used, a plaster cast was applied to provide sufficient stability to the implant. Full weight-bearing was allowed 10 weeks after surgery. Radiographic healing was usually evident by the end of the third month postoperatively.

**FIGURE 2:**
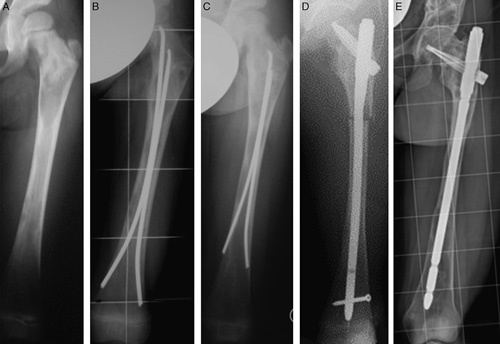
A, Type 1 femoral deformity in a 4-year-old girl affected by McCune-Albright syndrome causing a severe painful limp. B, One year after nailing with titanium elastic nails (TENs), pain had disappeared. C, Five years later, the painful limp recurred after marked femoral growth with relative shortening of the TENs that were removed and replaced with an adult cervicodiaphyseal nail (D). E, At the latest follow-up, 7 years after the third nail replacement, the patient was 21 years old and fully asymptomatic.

Titanium elastic nails (TEN: Synthes) were used in femurs or in tibias or in both bones of 6 children aged 4 to 8 years, while adult humeral nails with a spiral blade (PHN: Synthes) or adult interlocking humeral nails (UHN: Synthes) were used respectively in femurs and tibias of 4 children aged 6 to 9 years.^16^ In the remaining patients aged 9 years and over as well as in re-nailing of all our patients within this age range, an adult cervicodiaphyseal nail with a spiral blade (UFN: Synthes) was used in femurs^9^,^24^ while adult interlocking nails (UTN: Synthes or TN: Biomet), in tibias. There was no fixed protocol for timing of surgery or for using specific surgical techniques or specific implants: both fractures and the most symptomatic lower limb had IN priority. Moreover, both the surgical technique and the implant used were specifically tailored for the individual patient. No patient had fixation of more than one bone at the same surgical setting. IN was repeated during growth in two-thirds of the cases of the younger patients; thus, all the patients underwent a mean of 2.13 femoral (range: 3 to 1) and 1.50 tibial IN (range: 2 to 1) per limb (Fig. 2). Revision surgery was indicated when the distance of the extremities of the nail from the growth plates of the femur and tibia increased so that the unprotected bone was at risk of either fracture or deformity. Revision surgery was also indicated when one-half of the femoral neck became unprotected by the spiral blade or by TENs. At the time of nail replacement, rigid nails were easily removed as well as spiral blades. In contrast, it was usually difficult to remove TENs if they were totally incarcerated into the FD tissue of fully grown bones. Six femurs and 7 tibias had only 1 IN either because they were close to the end of growth or because the patients missed their scheduled follow-up. Mean age at the first IN was 9.26±2.68 years (range: 4 to 14 y) while at the last IN, 16.42±1.95 years (range: 11 to 19 y). On the basis of the extent of lower limb FD, 9 patients presented bimelic involvement and 12, monomelic. Fourteen cases required additional surgery (Table 1).

**TABLE 1:** Additional Surgery

Surgical Procedure	Number of Cases
Proximal valgus femoral osteotomy in infants for coxa vara correction stabilized with a 90 degrees proximal femoral plate (Synthes)	4 cases
*Distal medial femoral epiphysiodesis with staples (Wright) or 8 plates (Orthofix) for knee valgus correction	3 cases
*Proximal medial tibial epiphysiodesis with staples (Wright) or 8 plates (Orthofix) for knee valgus correction	2 cases
Distal medial tibial epiphysiodesis with 8 plates (Orthofix) for ankle valgus correction	2 cases
Distal femoral and proximal tibial epiphysiodesis with staples (Wright) or 8 plates (Orthofix) for lower limb length equalization	3 cases

* Wright staples were used before 2004; subsequently, Orthofix 8 plates were applied.

Of the 21 patients enrolled in the study, 17 came to the hospital for the latest follow-up, and they were evaluated with a clinical and radiographic scale (Table 2). Since this evaluation scale has never been used before, patients and their radiographs were evaluated by the 3 authors who were not directly involved in the patients’ surgical management (E.M., L.R., and G.G.). The correlation coefficients were as follows: intraobserver reliability was 0.815; interobserver reliability was 0.798. Four patients who lived far away were contacted by e-mail. They mailed our evaluation form filled out by their local orthopaedic surgeon, previously instructed by us, as well as their standing radiographs. Lower limb joints range of motion was measured with a hand goniometer. The radiographs done at follow-up were compared with those done after IN in order to score some of the radiographic parameters.

**TABLE 2:** Evaluation Scale of the Patients at Follow-up (A Modification of Guille et al^4^ and Jung et al^14^ Scales)

Clinical Parameters*	Radiographic Parameters*
1. Ambulation: normal gait; mild; moderate; severe limp	1. †Femoral and tibial shaft deformity on the coronal plane: <3 degrees; 3.1-5 degrees; 5.1-10 degrees; >10 degrees
2. Ambulatory aid: none; 1 cane; 2 canes or walker; wheelchair	2. ‡Mechanical axis deviation: <4 degrees;4.1-7 degrees; 7.1-10 degrees; >10 degrees
3. Shoe lift: none; 1-1.9 cm; 2-2.5 cm; >2.5 cm*	3. §Loss of the neck-shaft angle correction^5^: <5 degrees; 5.1-15 degrees; 15.1-20 degrees; >20 degrees
4. Pain on walking: absent; mild; moderate; severe	4. Lower limb length discrepancy: <1.5 cm; 1.5-2 cm; 2.1-3 cm; >3 cm
5. Climb stairs: possible; difficult; needing support; not possible	5. Knee height asymmetry: <2 cm; 2.1-3 cm; 3.1-4 cm; >4 cm
6. Run: possible; short distance; difficult; not possible	

* Four scores were assigned to each parameter: 0; 2.5; 5; and 10 points. In bimelic patients, only the worst score of the radiographic parameters 1, 2, and 3 measured in each lower limb was assigned to the overall score. An excellent result scored 110-90 points; good: 89-69; fair: 68-48; and poor: <48.

† Whether in valgus or in varus, deformity was expressed as the sum of the angular femoral deformities of both femur and tibia according to the Malalignment Test of Paley applied to the bowing deformities.^26^

‡ Whether in valgus or in varus, deviation was calculated according to Paley.^26^

§ Compared with the postoperative radiograph of the last corrective osteotomy performed before the follow-up evaluation.

Complications were classified using the Clavien–Dindo classification recognized by the American College of Surgeons. According to this classification, Grade IIIa complications require an intervention performed in local anesthesia while IIIb complications require either general or epidural anesthesia.

Descriptive statistics consisted of the mean±SD for parameters with normal distributions after confirmation with histograms and the Kolmogorov-Smirnov test. Comparisons between data were performed with the Student *t* test, the Fisher exact test, χ^2^ test, and analysis of variance test. Levels of significance reaching 95% or more were accepted, and a *P*-value of <0.05 was considered statistically significant.

## RESULTS

The mean age of the patients at the latest follow-up was 22.85±3.53 years (range: 14 to 29 y), and their lower limbs were fully grown in all but 1 patient. The mean length of follow-up from the last IN was 6.47±3.10 years (range: 3 to 14 y). The result of treatment and the average score of the patients are reported in Table 3. Monomelic patients scored better than bimelic ones (*P*<0.06); PFD monomelic, better than MAS monomelic (*P*<0.07), and the latter better than MAS bimelic (*P*<0.38). Intravenous administration of Zoledronic acid was stopped in the 8 patients who had previously had that treatment because their pain disappeared after IN, whereas calcitriol and phosphates treatment was continued after IN in the 7 patients with MAS.

**TABLE 3:** Age at Follow-up, Length of Follow-up, and Result of Treatment

Mean age at follow-up (and SD): 22.85±3.53 y (range: 14 to 29 y)	
Mean length of follow-up (and SD) since the last IN*: 6.47±3.10 y (range: 3 to 14 y)	
Monomelic: 12 patients (5 PFD and 7 MAS)	Bimelic: 9 patients (all MAS)
Excellent: 6 patients	Excellent: 2 patients
Good: 4 patients	Good: 2 patients
Fair: 1 patient	Fair: 2 patients
Poor: 1 patient	Poor: 3 patients
Mean score (and SD)	Mean score (and SD)
86.87±20.61 points	63.33±29.94 points
Mean score (and SD) of PFD patients	
97.91±9.27	
Mean score (and SD) of MAS patients	
75.83±23.59	

* Intramedullary nailing.

MAS indicates McCune-Albright syndrome; PFD, polyostotic fibrous dysplasia.

**FIGURE 3:**
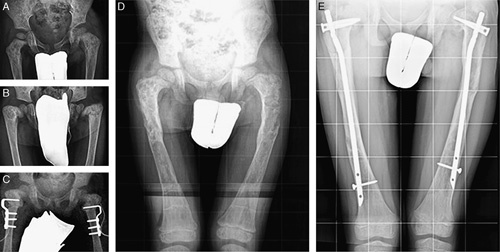
A, Patient with severe bilateral coxa vara with a painful waddling gait; bilateral valgus hip osteotomy (B) was performed. C, Standing radiograph at 6 years of age with type 4 femoral deformities. D, At 10 years of age, 4 years after bilateral femoral intramedullary nailing with adult humeral nails, bilateral coxa vara recurrence, and relative shortening of the nails with growth were evident (E).

**FIGURE 4:**
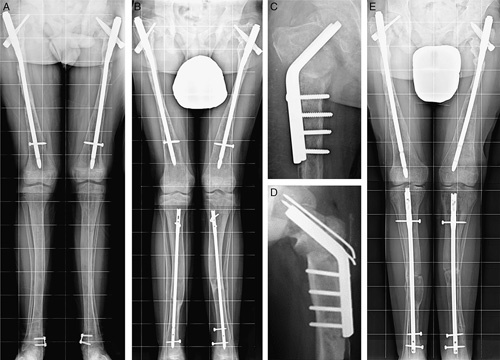
A, The same patient illustrated in Figure 3 at 11 years of age after intramedullary nailing (IN) with adult femoral nails and coxa vara correction. B, Two years later, after IN for fracture of both tibias. A severe coxa vara recurrence was treated by a 2-stage procedure (C, D) at the age of 14 years. E, At the latest follow-up, 4 years after the last IN, he had a lower limb length discrepancy of 1.5 cm and the mechanical axis was deviated in valgus of 3 degrees on the right and 5 degrees on the left. He walked with a slight limp without pain. Good result.

**FIGURE 5:**
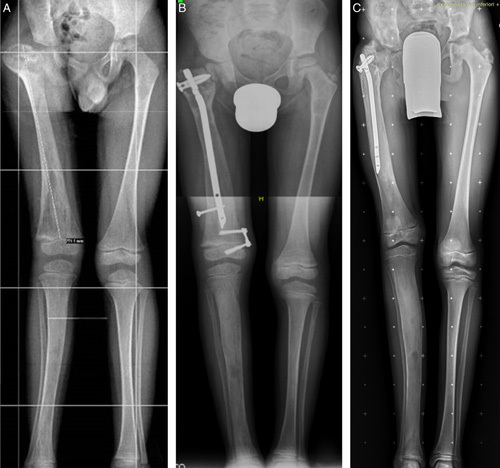
A and B, Five-year-old boy with McCune-Albright syndrome and monomelic involvement. He had a 50 degrees coxa vara and genu valgum, with a marked limp. C, Two years later, after a 2-stage correction of the femoral deformity and genu valgum correction by medial epiphysiodesis of the knee. When admitted for removal of both the loosened locking screw and the femoral 8-plate, the neck-shaft angle of the right femur measured 120 degrees and the right knee was straight. D, Lost for several years to follow-up, the patient accepted to participate in the study at the age of 14 years, showing deformities recurrence. He was the only patient with physes still open at the latest follow-up. Poor result.

**FIGURE 6:**
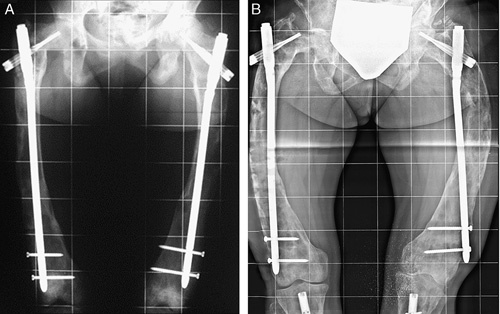
A, Standing anteroposterior radiograph of the pelvis and femurs of a 15-year-old patient with McCune-Albright syndrome, 2 years after her last bilateral intramedullary nailing. The patient was lost to follow-up but, 14 years later, she accepted to participate in the study. Radiographs (B) showed a severe recurrence of the femoral deformities with nail migration. She had a severe painful limp, but she was able to walk without any aid. Poor result.

At follow-up, 6 patients had a normal gait; 9, a mild limp; 3, moderate; 1, severe, and 2 used aids for walking. Pain was absent in 14 patients but still present in 7 when they were walking. Range of motion limitation was present in 9 hips, 4 knees, and 2 ankles. Mechanical axis deviation ranging from 3 to 12 degrees was present in 10 patients, while 8 patients lost from 10 to 40 degrees of the neck-shaft angle correction. Fourteen patients had lower limb length discrepancy ranging from 1 to 3.5 cm, and 6 of them wore a shoe lift. Six patients had a knee height asymmetry ranging from 1 to 4.5 cm (Figs. 2 and 3). The 4 patients who had a poor result had missed the scheduled follow-ups for several years, and in 3 of them the original nails had not been replaced (Figs. 4–6).

Complications occurred in 14 cases. Grade IIIb complications were: 1 subtrochanteric femoral nonunion with a broken UFN, healed after nail replacement, and autologous bone grafting; 1 femur refracture after TENs, healed with a hip spica cast application; 1 tibia refracture above an adult UHN 1 year after a double osteotomy, healed in plaster cast after nail removal; 2 femoral fractures below the plate following the first stage of a 2-stage procedure, healed after the second stage IN; 2 cases of acute postoperative loss of the neck-shaft angle correction after hip valgus osteotomy, successfully revised and stabilized with a postoperative spica cast application with the hip abducted; 1 femoral case of massive intraoperative bleeding requiring blood transfusions, with marked increase of the surgical time; 1 case of loosening of the cervical spiral blade caused by failure of the blade’s locking bolt, successfully revised. Grade IIIa complications were: 2 femoral and 3 tibial cases of late loosening of the nail distal locking screw, that was removed.

## DISCUSSION

Proper management since PFD/MAS’s early clinical manifestation is desirable in order to forestall morbidity and disability,^3^,^16^,^20^,^21^ as we did in our patients. We operated on 21 PFD/MAS patients whose femur and tibia were totally involved by FD tissue and who became candidates for surgery at an age ranging from 2 to 14 years. A total of 29 femurs and 14 tibias had IN that was repeated during growth in more than two-thirds of our cases. Additional surgery was also performed in 14 cases, mainly to fix genu valgum by guided growth and to equalize lower limbs length. As far as we know, this is one of the largest PFD/MAS pediatric case series treated with IN by the same surgical team, and with the longest follow-up ever reported.^3^,^8^,^12^,^15^–^17^,^19^–^21^,^23^,^24^ Since PFD is a genetic disease in which mutated and normal bone cells coexist in a mosaic arrangement,^1^,^6^ any attempt to eradicate FD tissue by curettage and bone grafting when a long bone is totally involved is not advisable because bone grafts will be resorbed and replaced by FD tissue.^3^,^6^,^7^,^10^,^21^,^27^ Similarly, curettage and bone cementing is contraindicated in children because polymethylmethacrylate may impair bone growth. Cortical strut grafts may be successful only in some monostotic forms.^1^ In our experience, intravenous bisphosphonates administration was beneficial for pain control in our PFD/MAS patients, but it did not improve bone strength since bone deformities progressed and fractures occurred even in the 8 patients treated with Zoledronic acid. Similarly, both calcitriol and phosphates administration did not fully normalize phosphoremia or the alkaline phosphatase serum level in any of the 7 patients with MAS.

We believe that the best treatment is to reinforce the affected bones by IN to prevent fracture and deformity that may occur until adulthood.^2^–^5^,^20^ However, the original nails must be replaced with bigger nails as children grow up, as already pointed out.^12^,^16^,^18^–^21^ Severe coxa vara ≤90 degrees, when present in types 3 and 6 femoral deformities, had a 2-stage correction because a too medial entry point of the nail in the cervicotrochanteric area—needed to correct such a severe deformity of the femoral neck—might cause either AVN of the femoral head by injuring the retinacular vessels or a fracture of the greater trochanter with loss of the implant stability. In those cases, a hip blade-plate was used to perform and stabilize the valgus osteotomy (first stage), while IN was performed 3 to 4 months later after hip plate removal (second stage).^24^ The self-elongating telescoping nails used in osteogenesis imperfecta appeared to be a solution. O’Sullivan and Zacharin^12^ treated 10 femurs and 1 tibia in 5 MAS children with Sheffield rods. However, their results were not satisfactory because at an average follow-up of 18 months coxa vara had recurred in 5 femurs and the fracture rate was not halted but only decreased. The main problems were the lack of femoral neck stabilization that may either worsen or cause coxa vara,^3^,^7^,^8^,^10^,^12^,^20^,^21^,^23^ and insufficient mechanical strength of the FD bone-rod construct because of the increase of bone size and body weight with growth. Hefti et al^19^ have proposed a new telescopic cervicodiaphyseal femoral nail, unusable in fractures because it is produced on a custom-made basis. Its preliminary results in deformity correction and stabilization at an average follow-up of 4.5 years are very encouraging. Peripheral plates frequently fail^3^,^8^,^10^,^16^,^19^,^23^ therefore, indications for plating should be very limited.^10^,^24^

Our patients underwent a mean of 2.13 femoral and 1.50 tibial INs. Both TENs and adult humeral nails were replaced with adult femoral and tibial nails in all the patients but those who were close to growth plate closure and those who missed their scheduled follow-up. On the basis of our experience, we do not recommend the routine use of TENs in bones totally affected by FD tissue because they require a long operative time and expose to high radiation doses as well as to maltracking and cutting-out owing to the lack of thick corticals to guide TENs’ progression along the diaphysis. Moreover, TENs—although pain-relieving—do not guarantee an absolute protection against refracture and deformity recurrence,^3^,^10^,^11^,^16^,^20^ and TENs’ removal may be difficult if they are fully incarcerated into the fibrodysplastic bone. Either the new generation of rigid femoral pediatric nails^28^ or the adult rigid humeral nails may be used as soon as they reach an appropriate fit in the femoral and tibial shaft.^16^ Therefore, TENs nailing should be limited to very young children.^20^

Fracture prevention was an important goal that was achieved by repeated IN. A high incidence of femoral fractures with a peak between 6 and 10 years has been reported in MAS.^5^ Therefore, our patients were helped very much by IN since many of them were nailed during the fracture age peak and only one femoral fracture recurred following TENs IN.

We only had 2 previously validated scales for measuring PFD outcomes,^13^,^18^ and we added to them additional scored clinical and radiographic parameters. The correlation coefficient values of our new scoring system reported in Methods appear to reflect good reproducibility and sufficient agreement among the observers. We judged the Enneking scale for bone tumors as not appropriate to evaluate a substantially benign condition like PFD.^1^

Monomelic patients fared better than bimelic ones, and PFD patients fared better than those with MAS. Both the extent of lower limb involvement and the hormonal influence that in MAS further worsens the poor quality of FD bone had a negative influence on our patients’ clinical outcome, as already reported.^1^,^3^,^4^,^6^,^10^ In fact, bone weakness of the upper femur may explain the loss of the neck-shaft angle—regardless of the support of the nail’s cervical component—that we observed in several MAS patients. However, the difference between bimelic and monomelic patients as well as between monomelic PFD and MAS patients was not statistically significant. This finding might be in part explained by the diffuse and severe involvement of both femur and tibia even in monomelic patients, that markedly affected their clinical result.

According to our scale, about 81% of our patients obtained satisfactory long-term results. Four MAS patients had a poor result, but all of them had missed their scheduled follow-ups for many years. Therefore, we recommend adherence to the scheduled follow-ups particularly in MAS patients because even a good result might deteriorate after the end of growth, as happened in one of our patients.^5^,^6^

IN presented 2 intraoperative problems, namely abundant bleeding and drilling a medullary canal through the FD bone.^3^,^9^,^12^,^15^,^20^,^24^ Preserving lower limb length equality was another problem.^3^,^17^,^21^ We performed epiphysiodesis but, at the latest follow-up, residual limb length discrepancy was still present in several patients though it was >2 cm in only 6 patients, who wore a shoe lift. Bone lengthening has been reported in PFD when corticals are thick enough to withstand a distraction apparatus.^29^ Unfortunately, corticals were very thin in our cases, thus not allowing lengthening.^3^,^20^,^21^

The overall incidence of complications—mainly affecting femoral IN and all requiring revision surgery—was high in our patients (32.5%). However, a high rate of complications was also reported by other authors who mainly treated children and adolescents with PFD/MAS.^7^,^12^,^17^–^20^,^23^ Surprisingly, Zhang et al^15^ reported no complications in 42 PFD patients under 18 years of age mostly treated by IN. In no case did we have infection^30^ and, as far as we know, only 3 cases of infection have been reported in the previous literature.^7^,^20^ The explanation for this remains speculative.

This study has several limitations. First, our cohort of patients treated by repeated IN lacks a control group. However, other protocols of treatment in children with PFD/MAS have not obtained better results than ours.^7^,^8^,^12^,^17^–^20^,^23^ Second, our study has a relatively short follow-up, since MAS patients are at risk of recurrence even after the end of growth, as happened in one of our patients. Third, our cohort included only a small number of patients. However, PFD and MAS are rare diseases, and we included only patients with total femoral and tibial involvement. Fourth, a new nonvalidated outcome scale, though partly made up of 2 previously validated scales and with a good intraobserver and interobserver reliability, was used to evaluate our patients. Last but not least, the treatment of our patients was not completely homogeneous: some adolescents had only 1 nailing whereas the youngest patients had multiple nailing. Moreover, the number of additional surgical procedures varied from case to case. This lack of homogeneity could have partly influenced our results.

In conclusion, we believe that IN—that must be repeated in younger children during growth—may be considered the best surgical treatment currently available in children when their lower limbs are severely affected by PFD. As far as we know, this is also the only study published on children with PFD/MAS surgically treated by the same team and followed up to the end of lower limb growth.
